# Use of the 2A Peptide for Generation of Multi-Transgenic Pigs through a Single Round of Nuclear Transfer

**DOI:** 10.1371/journal.pone.0019986

**Published:** 2011-05-13

**Authors:** Wei Deng, Dongshan Yang, Bentian Zhao, Zhen Ouyang, Jun Song, Nana Fan, Zhaoming Liu, Yu Zhao, Qinghong Wu, Bayaer Nashun, Jiangjing Tang, Zhenfang Wu, Weiwang Gu, Liangxue Lai

**Affiliations:** 1 Key Laboratory of Regenerative Biology, South China Institute for Stem Cell Biology and Regenerative Medicine, Guangzhou Institutes of Biomedicine and Health, Chinese Academy of Sciences, Guangzhou, China; 2 Institute of Comparative Medicine and Center of Laboratory Animals, Southern Medical University, Guangzhou, China; 3 College of Animal Science, South China Agricultural University, Guangzhou, China; Brunel University, United Kingdom

## Abstract

Multiple genetic modifications in pigs can essentially benefit research on agriculture, human disease and xenotransplantation. Most multi-transgenic pigs have been produced by complex and time-consuming breeding programs using multiple single-transgenic pigs. This study explored the feasibility of producing multi-transgenic pigs using the viral 2A peptide in the light of previous research indicating that it can be utilized for multi-gene transfer in gene therapy and somatic cell reprogramming. A 2A peptide-based double-promoter expression vector that mediated the expression of four fluorescent proteins was constructed and transfected into primary porcine fetal fibroblasts. Cell colonies (54.3%) formed under G418 selection co-expressed the four fluorescent proteins at uniformly high levels. The reconstructed embryos, which were obtained by somatic cell nuclear transfer and confirmed to express the four fluorescent proteins evenly, were transplanted into seven recipient gilts. Eleven piglets were delivered by two gilts, and seven of them co-expressed the four fluorescent proteins at equivalently high levels in various tissues. The fluorescence intensities were directly observed at the nose, hoof and tongue using goggles. The results suggest that the strategy of combining the 2A peptide and double promoters efficiently mediates the co-expression of the four fluorescent proteins in pigs and is hence a promising methodology to generate multi-transgenic pigs by a single nuclear transfer.

## Introduction

The genetic modification of swine can have a multitude of agricultural and medical applications. In agriculture, modifications in the swine genome could alter the pork composition and produce healthier meat, create swine strains that are more resistant to specific diseases, reduce the embryonic losses normally observed during the first month of swine embryogenesis, and breed animals that are more environmentally friendly [1]–[4]. In the medical field, specific genetic modifications in swine may afford organs for pig-to-human xenotransplantation, produce recombinant products for biomedical or nutraceutical use, as well as generate models of human diseases for medical research and drug development [3], [5], [6].

However, in many situations, multiple genes are required to be simultaneously modified in pigs to achieve a certain goal. For instance, in agriculture, it might be necessary to produce pigs with several favorable traits (e.g., low fat/muscle ratio, high disease resistance, and enriched with special nutrients), hence requiring multiple corresponding genes to be transferred into the pigs. In medical research, the transfer of multiple foreign genes into pigs is necessary for generating models of various human diseases, such as Alzheimer's disease and Parkinson's disease, which are related to multiple genes or factors [7], [8]. Multi-gene transfer has also been implicated in xenotransplantation research. Organs from multi-transgenic pigs expressing two or three complement genes involved in immunological rejection have exhibited prolonged xenograft survival [9]–[13].

Most multi-transgenic pigs have been produced by complex and time-consuming breeding programs that utilize different single-transgenic pigs [13], [14]. Sperm-mediated gene transfer (SMGT) has been previously reported for multi-transgenic pig production [15], but the prolonged expression of transgenes remains controversial [16]. A possible method to generate multi-transgenic pigs is by transfecting multiple plasmids (each containing one gene) simultaneously into porcine fetal fibroblasts (PFFs), which are then used for somatic cell nuclear transfer (SCNT). However, our previous data (unpublished) showed that the efficiency of this method was particularly low in both cell transfection and selection as well as in proteins co-expression in cloned piglets. The internal ribosomal entry site (IRES), which has been conventionally used for polycistronic expression cassettes, may be another choice for generation of multi-transgenic pigs. However, in vectors containing the IRES sequence, the gene placed downstream is often expressed at a much lower level than the gene located upstream [17], [18].

The viral 2A peptide has recently become an alternative to IRES for mediating polycistronic expression in gene therapy and somatic cells reprogramming [19]–[22]. This peptide, which originates from the picornaviruses, is approximately 19 amino acids long and contains a conserved and functional motif D (V/I) EXNPGP. It is also called a “self-cleaving” peptide or protease site because it is able to “cleave” at its own C terminus between the last two amino acids through ribosomal skipping during protein translation. Briefly, a normal peptide bond formation between the glycine and the proline is impaired at the 2A site, which causes the ribosome to skip and begin to translate from the second codon, resulting in the expression of two independent proteins from a single transcription event [23]. Considering the high “cleavage” efficiency at the 2A site, the 2A peptide can be used to mediate multiple genes expression in a polycistron. Previous studies have confirmed the feasibility of using the 2A site for the expression of multiple transgenes [24], [25], and 2A-based polycistronic expression has gained great success in generating induced pluripotent stem cells that require four factors working together [20], [21], [26]–[28]. The 2A peptide has also been shown to be more efficient in mediating multiple transgenes expression than IRES. Chinnasamy et al. [29] have reported that the expression of enhanced green fluorescent proteins (EGFP) in an MGMT-2A-EGFP bicistronic vector is approximately four times greater than that in an IRES-based vector. And the size of 2A peptide coding sequence is much smaller than that of IRES sequence. These unique properties of the 2A peptide make it a promising tool for generating multi-transgenic pigs.

In this study, a 2A peptide-based double-promoter expression vector that mediated the expression of four fluorescent proteins, namely, yellow fluorescent protein (ZsYellow1), enhanced cyan fluorescent protein (ECFP), red fluorescent protein (tdTomato), and EGFP was constructed to generate multi-transgenic pigs. Transgenic piglets were obtained, and the expressions of the four fluorescent proteins in these piglets were examined.

## Methods

### Ethics statement

The animal experiment was approved by the Department of Science and Technology of Guangdong Province, with an approval ID SYXK (Guangdong) 2005-0063, and complied with the guidelines of the Animal Care Committee, Guangzhou Institute of Biomedicine and Health, Chinese Academy of Sciences.

### Vector construction

The 2A sequence of the foot and mouth disease virus was synthesized and inserted into the pBlueScript KS(-) vector (pBS-2A). ZsYellow1, ECFP, tdTomato and EGFP, cDNAs were amplified by PCR from pZsYellow1, pECFPN1, pCMV-tdTomato, and pEGFPC1 (Clontech), respectively, and cloned into pBS-2A to generate pBS-cDNA-2A vectors. The tdTomato-2A and ZsYellow1-2A fragments were then cut off from the corresponding vectors and inserted into pBS-EGFP-2A to generate pBS-tdTomato-2A-EGFP-2A-ZsYellow1-2A.The tdTomato-2A-EGFP-2A-ZsYellow1-2A construct was then ligated to ECFP cDNA and inserted into a pCAG vector containing the CAG promoter (a composite of chicken β-actin promoter and cytomegalovirus early enhancer element) [30] to generate pCAG-tdTomato-2A-EGFP-2A-ZsYellow1-2A-ECFP (pTGZC).

The EGFP and ECFP cDNAs were inserted into pBS-tdTomato-2A and pBS-ZsYellow1-2A generating pBS-tdTomato-2A-EGFP and pBS-ZsYellow1-2A-ECFP, respectively, to construct a vector in which the expressions of the four fluorescent genes were driven by two independent CAG promoters. The tdTomato-2A-EGFP (TG) and ZsYellow1-2A-ECFP (ZC) constructs were then subcloned into the pCAG vector to generate pCAG-TG and pCAG-ZC, respectively,followed by the insertion of the CAG-ZC fragment into the pCAG-TG vector to generate the 2A peptide-based double-promoter vector pZCpTG.

### Primary cell isolation and culture

PFFs were prepared as previously described [5]. Briefly, A 35-day-old fetus (landrace) without head, limbs, and internal organs were minced in phosphate buffered saline (PBS) and digested in Dulbeccos' modified Eagle's medium (DMEM) supplemented with 15% fetal bovine serum (FBS) (Hyclone), 1% penicillin-streptomycin (Gibco), 0.32 mg/ml Collagenase IV (Sigma), and 2500 IU/ml DNase I for 6 h at 39°C. The cells were then centrifuged at 1,000 rpm for 5 min, followed by suspension in DMEM supplemented with 15% FBS, 0.5% penicillin-streptomycin, 5% l-glutamine, 2.5% pyruvate, and 2.5 ng/ml basic fibroblast growth factor. The cells were cultured in 10 cm dishes for 12 h and then frozen in FBS containing 10% dimethyl sulfoxide for future use.

Ear tissues from newborn piglets were treated with 75% ethanol for 5 min and then washed thrice with PBS containing 1.5% penicillin-streptomycin. The ear tissues were minced, and the fibroblasts in the ear were isolated and cultured in the same manner as described above.

### PFFs transfection and selection

PFFs were thawed and cultured in 10 cm dishes until 90% confluent. Then, 1×10^6^ PFFs in 800 µl PBS containing 25 µg of linearized pTGZC or pZCpTG were electroporated at 230 v/cm and 500 µF using a Gene Pulse Xcell electroporator (Bio-Rad). After 24 h of recovery, the electroporated cells were selected with 1 mg/ml G418 (Merck) for 10 days. Cell colonies co-expressing the four fluorescent proteins were picked up and cultured in 48-well plates. The cells were transferred to 35 cm dishes and cultured for 3–4 days until confluent, and then the cells were frozen for future use.

### SCNT and transgenic pig generation

Oocytes were processed as previously described [5]. Pig ovaries were purchased from a local slaughter house. Briefly, cumulus oocyte complexes (COCs) were aspirated from antral follicles and washed with maturation medium (Tissue Culture Medium 199) (Gibco) supplemented with 0.1% (w/v) polyvinyl alcohol (Sigma), 3.05 mM d-glucose, 0.91 mM sodium pyruvate (Sigma), 0.57 nM cysteine (Sigma), 0.5 µg/ml LH (Sigma), 0.5 µg /ml FSH (Sigma), 10 ng/ml epidermal growth factor (Sigma), 10% (v/v) porcine follicular fluid, 75 µg /ml penicillin G, and 50 µg /ml streptomycin. The COCs were then transferred to a four-well multidish (Nunc) containing 500 µl of the maturation medium covered with mineral oil and pre-equilibrated at 39°C overnight. After 42–44 h of incubation, the oocytes were released from the COCs by vigorous vortex for 4 min in TL-Hepes containing 0.1% polyvinyl alcohol and 0.1% hyaluronidase (Sigma). The polar body and associated metaphase plate of the oocytes were aspirated using glass pipettes in micromanipulation medium supplemented with 7.5 µg/ml cytochalasin B. Denuded oocytes were used as the recipients for SCNT.

The transgenic PFFs were thawed and grown to subconfluency prior to SCNT. Cells with uniform co-expression of the four fluorescent proteins were selected under fluorescent microscopy and used as donor cells that were injected into the perivitelline space of the oocytes. The oocyte-donor cell complexes were activated to fuse and become reconstructed embryos with two successive DC pulses at 1.2 kv/cm for 30 µs using an electrofusion instrument (BLS). The reconstructed embryos were cultured in embryo-development medium PZM3 at 39°C overnight and then surgically transferred to recipient gilts exhibiting natural estrus. The pregnancy status of the gilts was monitored weekly using an ultrasound scanner beginning on Day 24 after the implantation. All cloned piglets were delivered by natural birth. To monitor in vitro development, transgenic and nontransgenic SCNT embryos were cultured for 6 days until they reached the blastocyst stage.

### Genotype identification

Genomic DNA was extracted from primary fibroblasts isolated from newborn piglets using PureLink Genomic DNA Mini Kit (Invitrogen). Two pairs of primers were designed to detect ZC and TG fragments, with each primer located in the coding sequence of each fluorescent gene. The ZC forward (5′-CATGTACCTGCTGCTGAAG-3′) and reverse (5′-CTGAAGCACTGCACGCCCCAG-3′) primers yielded a 507 bp amplification product. Given that tdTomato cDNA contains two tandem repeated sequences and the TG forward primer is located in this sequence, two products (1572 bp and 488 bp) were amplified with the TG forward (5′-GTGGAGTTCAAGACCATCTAC-3′) and reverse (5′-CTGAAGCACTGCACGCCGT-3′) primers. For PCR, 0.1 µg of genomic DNA extract was added to 19 µl PCR buffer containing 0.2 mM primers, 0.2 mM dNTPs, 50 U/ml of ExTaq polymerase (TaKaRa). The pZCpTG vector DNA was used as positive control, whereas the genomic DNA of wild-type (WT) PFFs was used as negative control. The PCR products were analyzed by 2% agarose gel electrophoresis.

### Fluorescent protein expression analysis

Fibroblasts were seeded on coverslips for 24 h, fixed with 4% paraformaldehyde for 5 min, washed with PBS, and mounted onto slides for observation. One of the transgenic piglets was sacrificed, and various tissues, including the liver, kidney, hoof, heart, tongue, skin, nose, and lung, were fixed in 4% paraformaldehyde at 4°C overnight, washed thoroughly with PBS, and cryosectioned at 20 µm thickness. The fibroblasts and tissue slices were observed under a confocal microscope (Leica TCS SP2). ZsYellow1, ECFP, tdTomato, and EGFP were excited at 514, 458, 543, and 488 nm, respectively. Fluorescence intensity was measured using Leica Confocal Software.

The co-expression of the fluorescent proteins in the reconstructed embryos was observed using a fluorescence microscope (Olympus BX51) under appropriate excitation filters (490–500 nm for ZsYellow1, 425–445 nm for ECFP, 535–555 nm for tdTomato, and 460–480 nm for EGFP).

The fluorescent proteins expressed in the living transgenic piglets were directly observed using goggles specifically designed by Biological Laboratory Equipment Maintenance and Service Ltd. for observing fluorescence in living organisms.

### Real-time PCR

Total RNA was extracted from various tissues of transgenic piglets using TRIzol Reagent (Invitrogen) and treated with DNase I (TaKaRa) according to the provided instructions. First-strand cDNA was synthesized using Superscript™ III Reverse Transcriptase (Invitrogen), and mRNA levels of target genes were determined by real-time PCR. The real-time primers for ZsYellow1 were: forward (5′-CCCAGGACATCGTGGACTACTT-3′) and reverse (5′-ACGCCGTTGAAGATGCTCTT-3′), whereas those for tdTomato were: forward (5′-GCTGAAGGGCGAGATCCA-3′) and reverse (5′- GTGGGAGGTGATGTCCAGCTT-3′). Considering that only a few nucleotides are differed between the coding sequences of EGFP and ECFP, we designed a primer pair that was able to detect both EGFP and ECFP: forward (5′-CATGCCCGAAGGCTACGT-3′) and reverse (5′- GCTTGTGCCCCAGGATGTT-3′). 18S rRNA was used as the reference gene.

### Statistical analysis

Data in this study were expressed as mean ± S.D. of at least three independent experiments. Statistical significance was determined using the two-tailed Student's *t*- test. *p*<0.05 was considered statistically significant.

## Results

### Construction of vectors for the co-expression of four fluorescent proteins

**Figure 1:**
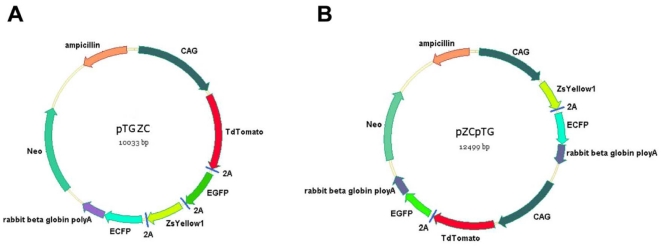
Maps of the pTGZC (A) and pZCpTG (B) vectors.

The pTGZC and pZCpTG vectors were constructed via multiple steps of cloning as described in Methods, and their maps were shown in Figure 1. The pTGZC vector contained a polycistronic cassette TGZC, which was mediated by the 2A peptide. The transcription of TGZC was driven by a single CAG promoter. In contrast, the pZCpTG vector contained two CAG promoters and two independent bicistronic cassettes: TG and ZC. Each cassette has its own CAG promoter. The pTGZC and pZCpTG vectors were transfected into 293T cells using Lipofectamine 2000 (Invitrogen). The uniformly high co-expression levels of the four fluorescent proteins showed that both vectors worked well in cell lines (data not shown).

### Co-expression of the four fluorescent proteins in PFFs and blastocysts

**Figure 2:**
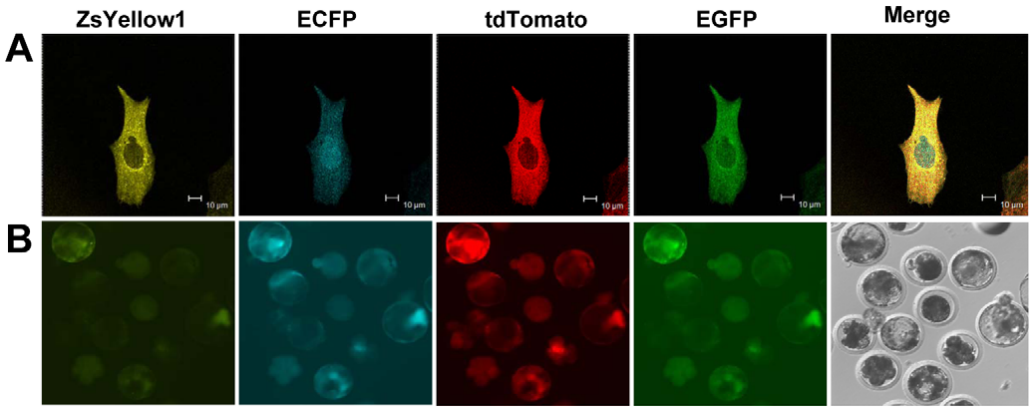
Co-expression of the four fluorescent proteins in PFFs and blastocysts. (A) PFFs transfected with the linearized pZCpTG vector were observed under a confocal microscope using appropriate filters. The scale bar represents 10 µm. (B) Several reconstructed embryos derived from the pZCpTG PFFs were cultured until they reached the blastocyst stage. The blastocysts were observed under a fluorescence microscope using appropriate filters.

Ten days after G418 selection, 10 PFFs colonies transfected with linearized pTGZC were randomly selected from each of the eight 10 cm dishes to evaluate the co-expression of the four fluorescent proteins in PFFs. Among the 80 selected colonies, 19 (23.75%) were found to co-express the four proteins and only 6 (7.5%) co-expressed all four proteins at uniformly high levels. In the SCNT-blastocysts derived from the pTGZC PFFs, the fluorescence intensities of the proteins encoded by the downstream genes (ZsYellow1 and ECFP) were markedly lower than those of the proteins encoded by the upstream genes (tdTomato and EGFP) in the vector (data not shown). However, when the 2A-based double-promoter vector pZCpTG was used, 54.3% (38/70) of the colonies obtained from the seven dishes co-expressed the proteins at uniformly high levels (Figure 2A). The blastocysts derived from the pZCpTG PFFs also consistently co-expressed the four fluorescent proteins (Figure 2B).

These results suggest that although the 2A peptide was highly efficient in mediating polycistronic expression (pTGZC) in the cell lines, it was not significantly efficient in the primary cells and embryos, especially for the expression of the downstream genes. However, when it was used solely for mediating a bicistronic expression (pZCpTG), it was efficient in the primary cells and embryos. Therefore, the 2A-based double-promoter vector pZCpTG was used for the generation of the multi-transgenic pigs in this study.

### *In vitro* development of multi-transgenic embryos

The rate of embryonic development *in vitro* was compared between transgenic SCNT (pZCpTG-PFF) and nontransgenic SCNT (WT-PFF) embryos (Table 1). The overall developmental rates of transgenic and nontransgenic SCNT embryos showed no significant differences at both two-cell stage [(86.8±0.8)% vs. (87.9±1.0)%] and blastocyst stage [(20.4±1.3)% vs (20.9±1.0)%], suggesting that the transgenes had no significant impact on embryonic development.

**Table 1:** Development ability of nuclear transfer embryos derived from pZCpTG-PFFs and WT-PFFs.

Embryo type	Cultured	Cleaved (%)	Blastocyst (%)
WT-PFF	235	206 (87.9±1.0)	47 (20.9±1.0)
pZCpTG-PFF	240	208 (86.8±0.8)*	43 (20.4±1.3)*

The embryo numbers are the total numbers counted from three independent experiments, and the percentage values in the third and fourth columns are shown as mean ± S.D.

*p>0.05 compared with WT-PFF; no significant difference.

### Generation of multi-transgenic pigs and genotype identification

The pZCpTG PFFs co-expressing the four fluorescent proteins were used as donor cells for SCNT to obtain multi-transgenic pigs. A total of 840 reconstructed embryos were transferred to 7 recipient gilts. Early pregnancy was observed in 4 gilts, and 2 gilts went to full term. Eleven live male piglets were born from the 2 gilts by natural delivery. These piglets appeared normal at birth and were observed to have normal growth 8 months later.

**Figure 3:**
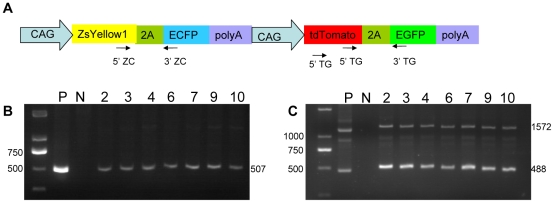
Genotype identification of multi-transgenic piglets. (A) Positions of the two primer pairs for genotype identification. Genomic DNA from Piglet 2, 3, 4, 6, 7, 9, 10 was extracted, whereas ZC (B) and TG (C) bicistronic cassettes in the genome were detected by PCR using appropriate primers. Lane 1, DNA marker; lane 2, positive control; lane 3, negative control; lane 4–10, genomic DNA from Piglet 2, 3, 4, 6, 7, 9 and 10.

Genomic DNA extracted from the ear fibroblasts of Piglets 2, 3, 4, 6, 7, 9 and 10 was used for PCR to confirm the presence of the four fluorescent protein genes in the genome of the cloned piglets. As shown in Figure 3, these piglets contained the four fluorescent protein genes, confirming that they were multi-transgenic pigs.

### Co-expression of the four fluorescent proteins in fibroblasts isolated from multi-transgenic piglets

**Figure 4:**
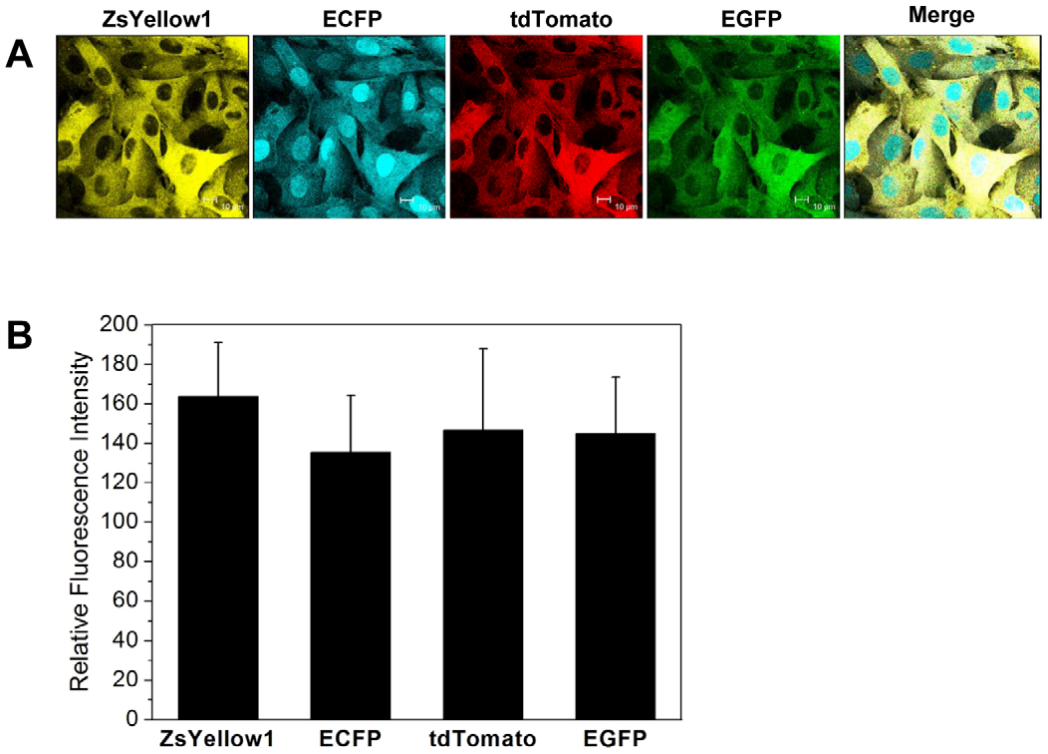
Co-expression of the four fluorescent proteins in the fibroblast isolated from multi-transgenic piglets. (A) Fibroblasts isolated from the ear tissue of a representative animal (Piglet 10) observed under a confocal microscope using appropriate filters. The scale bar represents 10 µm. (B) The fluorescence intensities of the four fluorescent proteins were measured. The results are shown as mean ± S.D.

The fibroblasts isolated from the ear tissue of the newborn piglets were observed under a confocal microscope, and the co-expression of the four fluorescent proteins was observed in fibroblasts from all of eleven piglets. The fibroblasts from most multi-transgenic piglets, including Piglet 2, 3, 4, 5, 7, 10, and 11, exhibited excellent co-expression of all proteins. Slight variations of protein expression were observed in the fibroblasts from Piglet 1, 6, 8, and 9. As shown in Figure 4, the fluorescence intensities of all proteins were identical in fibroblasts from Piglet 10.

### Co-expression of the four fluorescent proteins in various tissues of a multi-transgenic piglet

**Figure 5:**
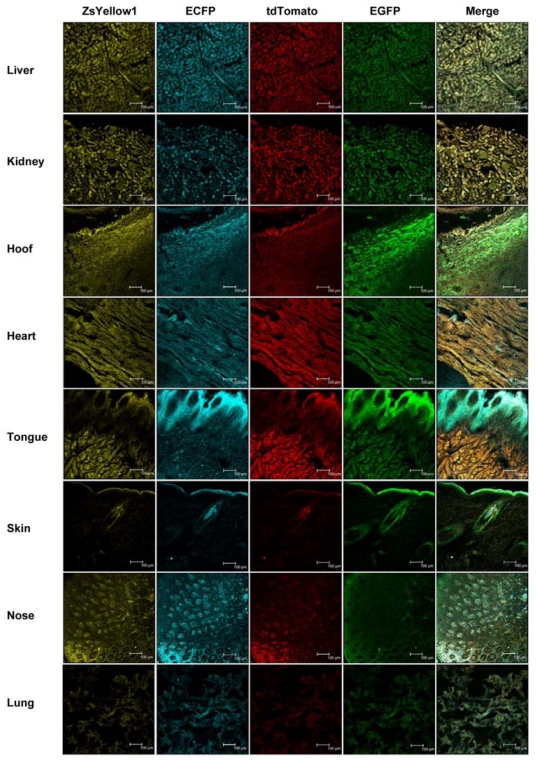
Co-expression of the four fluorescent proteins in various tissues of a multi-transgenic piglet. Liver, kidney, hoof, heart, tongue, skin, nose and lung tissues from Piglet 7 were cryosectioned and observed under a confocal microscope using appropriate filters. The scale bar represents 100 µm.

Piglet 7 was sacrificed and the major tissues, including the liver, kidney, hoof, heart, tongue, skin, nose, and lung, were collected for expression analysis of the four fluorescent proteins. Different fluorescent proteins exhibited rather consistent and uniform expression in the same tissue (Figure 5), but the protein expression levels in the different tissues slightly differed, possibly due to the tissue specificity of the promoter and the protein synthesis activities of the different tissues.

**Figure 6:**
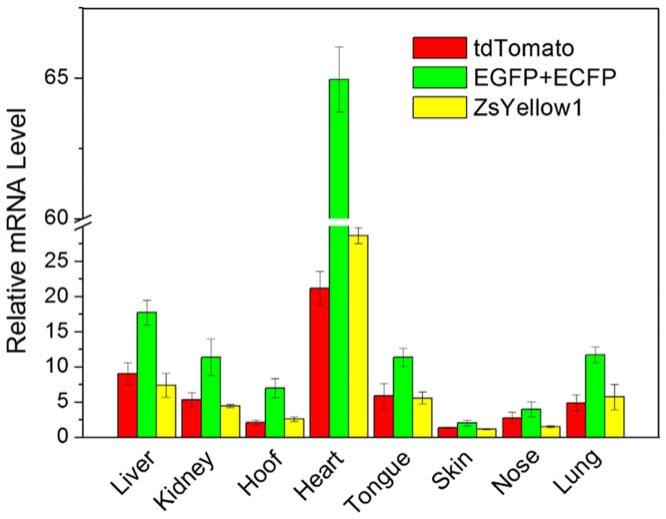
Expression profile analysis of four fluorescent proteins in various tissues by RT-PCR. Total mRNA was extracted from various tissues of Piglet 7. The mRNA levels of the target genes (tdTomato, EGFP+ECFP and ZsYellow1) were determined by RT-PCR. The results are shown as mean ± S.D.

Real-time PCR was introduced to detect the mRNA levels of the different proteins in the above tissues to confirm the uniform co-expression of the four fluorescent proteins further. However, considering that the coding sequences of EGFP and ECFP showed extremely high similarity (99%), we could only design a pair of primers that was able to detect both EGFP and ECFP. As shown by the real-time PCR results in Figure 6, the EGFP+ECFP mRNA level were always approximately 2 folds of tdTomato and ZsYellow1 mRNA level in the eight tissues. An especially high mRNA expression was found in the heart tissue, whereas the mRNA levels in other tissues were relatively close. This data suggest that different fluorescent genes have consistent expression in the same tissue, and the CAG promoter had an extremely high activity in the heart.

### Observation of fluorescence in living multi-transgenic piglets

**Figure 7:**
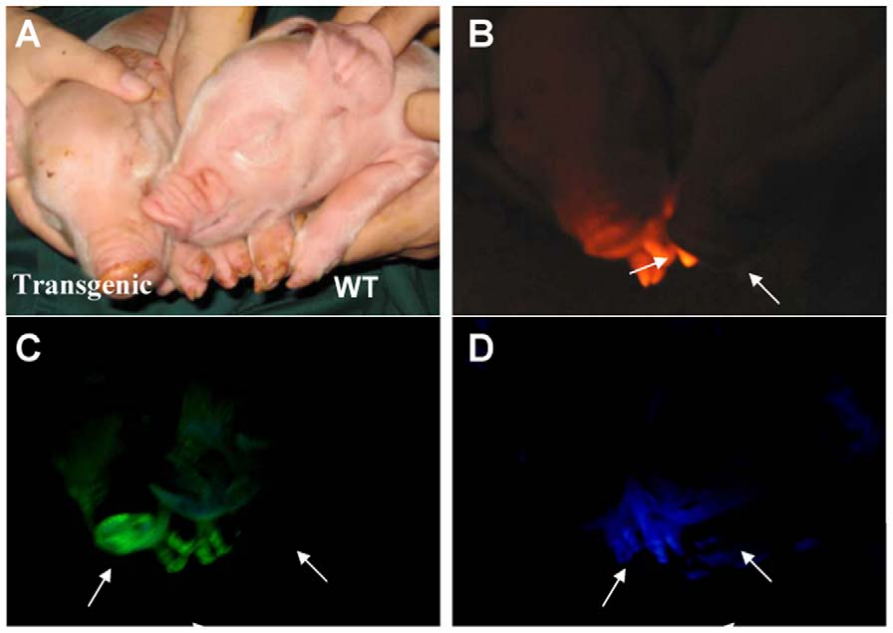
Fluorescence in living multi-transgenic piglets. Florescence on the noses and hooves of multi-transgenic piglets was directly observed using goggles. (A) Bright light. (B) tdTomato fluorescence. (C) EGFP fluorescence. (D) ECFP fluorescence. A WT piglet was used as negative control.

Fluorescent protein expression in living piglets was then evaluated using goggles. With appropriate excitation and emission filters, high levels of tdTomato, EGFP, and ECFP fluorescence were observed in the nose, hoof, and tongue of the multi-transgenic piglets, but not in the control piglet (Figure 7), further supporting the high expression of fluorescent proteins in the piglets. However, the instrument used was not sensitive enough to discern yellow fluorescence, whose emission wavelength is extremely close to that of EGFP. High ZsYellow1 expression in multi-transgenic piglets has already been confirmed by confocal microscopy observation and real-time PCR.

## Discussion

Expanding applications of transgenic pigs, especially multi-transgenic pigs, have been observed in agricultural and medical research. Production of multi-transgenic pigs, however, remains to be a great challenge. In this study we have underlined an effective method for generating multi-transgenic pigs via a single round of SCNT using the 2A peptide-based double-promoter vector pTGpZC. All 11 piglets we obtained were multi-transgenic pigs, with 7 piglets co-expressing the four fluorescent proteins at equivalently high levels and 4 showing slight discrepancy in protein expression.

Several studies on the generation of multi-transgenic pigs have been previously reported. Zhou et al. [13] successfully produced triple-transgenic pigs co-expressing human CD59, human membrane cofactor protein (hMCP), and human decay-accelerating factor (hDAF) by breeding among single-transgenic pigs. However, different single-transgenic pigs should first be produced with their method, which is time-consuming and costly. The combination of all transgenes is not necessarily achieved if the single-transgenic pigs are heterozygous. In addition, the prolonged period of sexual maturity and gestation of pigs requires substantial time to generate multi-transgenic pigs under this method.

SMGT, another technique in which sperms are pre-incubated with multiple transgenes before insemination, has also been optimized to produce multi-transgenic pigs [15]. Although this method is of low cost, requires a short time to complete, and is easy to use, the gene insertion is random, which might yield rather different expression levels for different genes in the piglets [16]. In addition, the sperms with all transgenes could not be intentionally selected for artificial insermination, which results in the low efficiency of multi-transgenic pig generation. The transgenes introduced by SMGT have also been reported to be transiently transmitted to the offspring at the early growth stage and be most likely lost in adulthood [31].

Compared with the aforementioned strategies, our proposed method has the following advantages: (1) It only requires a single round of nuclear transfer and saves significant time and money. (2) The four fluorescent genes are most likely to stay together and exhibit a “one-expression, all-expression” pattern. (3) The expression levels of the four fluorescent genes are consistent due to their uniform conditions in the genome along with the two CAG promoter-driven bicistronic cassettes and the high “cleavage” efficiency of 2A. This consistence in expression levels of the 2A upstream and downstream genes would probably be crucial to the application of multi-transgenic pigs. (4) The donor cells for SCNT are selected by G418, enabling to integration of the four genes into the genome, resulting in a stable expression of transgenes throughout the whole lifetime of transgenic pigs. (5) Cells with a uniformly high co-expression of the four fluorescent proteins could be selected directly under fluorescent microscopy and used as donor cells for SCNT, which significantly increases the efficiency of multi-transgenic pig production. (6) This method could be easily applied to other functional genes. Three fluorescent genes in our vector could be replaced with three functional genes, with one fluorescent gene being retained as a marker for donor cell selection prior to SCNT and protein expression in transgenic piglets.

The 2A peptide-based single-promoter vector pTGZC, in which the four fluorescent genes linked by 2A peptide were driven by a single CAG promoter, was also constructed in this study. However, it was not efficient enough to mediate the co-expression of the four genes in the PFFs and embryos. The expression of the last two genes was much weaker than that of the first two genes in the polycistronic cassette, suggesting that the “cleavage” efficiencies at the downstream 2A sites were lower than those at the upstream 2A sites. Thus, we chose the double-promoter vector containing two 2A-mediated bicistronic cassettes for multi-transgenic generation. Nevertheless, the mechanism for the increased incomplete “cleavage” at the downstream 2A sites remains to be elucidated.

The “cleavage” at a 2A site results in an upstream protein fused with the 2A peptide without proline residue and a downstream protein with a proline residue added to the N terminus. The addition of a single proline to the N terminus of the second protein should not significantly affect its function. However, whether the addition of the 2A peptide to the first gene product does not interfere with its intracellular localization and function in transgenic pigs need to be verified. Most studies have shown that the intracellular trafficking [19], [29] and function [32], [33] of the transgene products are not affected by the 2A fusion, but mistargetting [34] and functional abrogation [35] of transgene products caused by such fusion have also been reported. Two strategies may possibly address this problem in 2A-mediated multi-transgenic production. First, the co-expression cassette could be tested to ensure that all transgene products have normal localizations and functions in donor cells before SCNT. The gene that might be affected by 2A addition could be placed downstream of the 2A site. Second, the furin cleavage site could be introduced to remove 2A-derived amino acid residues *in vivo* [36]. Furin, an endogenous cellular proprotein convertase, could cleave at the cleavage site sequence RXK/RR [37], [38]. Therefore, placing a furin cleavage site between the upstream gene and the 2A sequence would eliminate all possible adverse effects caused by the remaining 2A residues [36].

In this study, the mRNA levels of the four transgenes were much higher in the heart than in other tissues due to the high activity of the β-actin promoter-based CAG promoter in this organ, which is rich in actin filaments [39]. The especially high expression of transgenes driven by the CAG promoter has also been observed in the mouse heart [40], [41]. In the present work, the CAG promoter was selected to construct a multi-transgenic pig model. In other practical applications numerous promoters could be used. For example, the liver-specific human apolipoprotein E promoter could drive transgene expression in a liver-specific pattern [42], which would be especially useful when the multi-transgenic pigs are used as liver donors in xenotransplantation and the transgene products are detrimental to other tissues in the pig.

In summary, multi-transgenic pigs uniformly co-expressing four fluorescent proteins in various tissues were successfully produced using a 2A peptide-based double-promoter vector through a single round of SCNT. This study demonstrates that the 2A peptide could be used to generate multi-transgenic pigs with high efficiency, and provides a promising methodology to generate multi-transgenic large animals.
